# A Model for the Early Identification of Sources of Airborne Pathogens in an Outdoor Environment

**DOI:** 10.1371/journal.pone.0080412

**Published:** 2013-12-04

**Authors:** Jeroen P. G. van Leuken, Arie H. Havelaar, Wim van der Hoek, Georgia A. F. Ladbury, Volker H. Hackert, Arno N. Swart

**Affiliations:** 1 Institute for Risk Assessment Sciences (IRAS), Utrecht University, Utrecht, The Netherlands; 2 Centre for Infectious Disease Control (CIb), National Institute for Public Health and the Environment (RIVM), Bilthoven, The Netherlands; 3 University of Glasgow, Glasgow, United Kingdom; 4 Municipal Health Service Zuid-Limburg, Sittard-Geleen, The Netherlands; The Pirbright Institute, United Kingdom

## Abstract

**Background:**

Source identification in areas with outbreaks of airborne pathogens is often time-consuming and expensive. We developed a model to identify the most likely location of sources of airborne pathogens.

**Methods:**

As a case study, we retrospectively analyzed three Q fever outbreaks in the Netherlands in 2009, each with suspected exposure from a single large dairy goat farm. Model input consisted only of case residential addresses, day of first clinical symptoms, and human population density data. We defined a spatial grid and fitted an exponentially declining function to the incidence-distance data of each grid point. For any grid point with a fit significant at the 95% confidence level, we calculated a measure of risk. For validation, we used results from abortion notifications, voluntary (2008) and mandatory (2009) bulk tank milk sampling at large (i.e. >50 goats and/or sheep) dairy farms, and non-systematic vaginal swab sampling at large and small dairy and non-dairy goat/sheep farms. In addition, we performed a two-source simulation study.

**Results:**

Hotspots – areas most likely to contain the actual source – were identified at early outbreak stages, based on the earliest 2–10% of the case notifications. Distances between the hotspots and suspected goat farms varied from 300–1500 m. In regional likelihood rankings including all large dairy farms, the suspected goat farms consistently ranked first. The two-source simulation study showed that detection of sources is most clear if the distance between the sources is either relatively small or relatively large.

**Conclusions:**

Our model identifies the most likely location of sources in an airborne pathogen outbreak area, even at early stages. It can help to reduce the number of potential sources to be investigated by microbial testing and to allow rapid implementation of interventions to limit the number of human infections and to reduce the risk of source-to-source transmission.

## Introduction

Several airborne infectious diseases, including Q fever, foot-and-mouth disease, legionellosis, and avian influenza, have outdoor sources and no (significant) human-to-human transmission. When an outbreak of such a disease occurs, it is a public health priority to identify the location of the source(s) as soon as possible in order to be able to implement control measures. Taking (air) samples from putative sources or their nearby surroundings can be helpful, but collecting and analysing these samples can be time-consuming and expensive. Atmospheric dispersion models (e.g., [1]) have been used for modeling of airborne transmission of, among others, the foot-and-mouth disease virus (e.g., [2]), Legionella bacteria (e.g., [3]), and *Coxiella burnetii* (Q fever) (e.g., [4]). However, they require a known source location, whereas in outbreak control a reverse approach – to identify the source by means of the notified cases – would be more helpful. Therefore, we developed a model to detect the source of outbreaks of airborne pathogens, using only data on human population density, case residential addresses and day of onset of clinical symptoms.

As a case study, we used data from three large regional Q fever outbreaks in the Netherlands that occurred in 2009 [5]. Q fever is an airborne infectious disease [6], caused by the gram-negative bacterium *Coxiella burnetii*, and human infection occurs mainly by inhalation of contaminated aerosols [7]. During the Dutch Q fever epidemic – from 2007 through 2010 – outbreaks were generally associated with dairy goat farms and to a lesser extent with dairy sheep farms [8]. The authorities needed much time to identify the farms that were responsible for the human infections. Farms were designated Q fever positive based on either (A) a Q fever-induced abortion rate >5% [9], and/or (B) a non-systematic bulk tank milk (BTM) program performed in the autumn of 2008 [10], and/or (C) a systematic BTM monitoring program mandatory from September 2009 [8], and/or (D) positive vaginal goat or sheep swabs [11], [12].

From December 2009, all dairy goats were vaccinated and all pregnant dairy goats on positive farms with >50 goats were culled [13]. The number of cases subsequently dropped sharply in 2010. If the authorities however had the ability to use a model as a first indicator for farm infections, then costs (time/money), and the number of human infections could have been lower [14].

## Methods

### Data

The current study was limited to the year 2009. We selected three Q fever case cluster, each with a single positive large dairy goat farm as the suspected source: the center of Utrecht province (area A) [5], the southeast of Noord-Brabant province (area B) [5], [15], and the south of Limburg province (area C) [5], [16]. Population density data was available at the six-digit zip code level (PC6, i.e. street-level). Data of cases notified in 2009, including the residential addresses at the PC6-level and dates of disease onset, were available from the Municipal Health Services (MHS) of Utrecht & Midden-Nederland (n = 120), Brabant-zuidoost (n = 367), and Zuid-Limburg (n = 230). Dutch legislation allows using this case information for research purposes if information is not traceable to individual patients. In this case, consent of cases is not required. The case information can however not be made publicly available.

Information on all goat and sheep farms in the Netherlands was made available by the Ministry of Economic Affairs, Agriculture and Innovation (used for validation). It includes the exact location of all goat/sheep farms and the number of goats/sheep per farm in November 2009.

Q fever status was available for all dairy and some non-dairy farms. In 2008, the Animal Health Service performed a non-systematic BTM program at approximately 66% of the large dairy goat farms (>50 goats) [10]. From September 2009, the Netherlands Food and Consumer Product Safety Authority implemented a mandatory BTM monitoring program for all large dairy goat farms [8]. Abortion rates of >5% [9], indicative for Q fever [8], [17], were reported by a number of farms and available from the Animal Health Service. Finally, vaginal swabs were taken at a selected number of large and small dairy and non-dairy goat and sheep farms [8], [18].

In each outbreak area (A, B and C) there was a single large dairy goat farm being Q fever positive based on at least one of the above criteria and therefore considered as the suspected source. The suspected farm in area A (1295 goats) was positive in the 2008 and 2009 BTM program, and vaginal swabs from goats at this farm were positive in July 2009. The farm in area B (791 goats) reported an abortion wave in April 2008, was positive in the 2008 and 2009 bulk tank milk program, and had positive vaginal swabs in May 2009. The farm in area C (1135 goats) reported an abortion wave in March 2009 and was positive in the 2008 and 2009 BTM program (no vaginal swabs were taken here). Based on these results, we assumed the suspected goat farms were infectious during the complete study period. No other large goat/sheep farms in the case cluster areas reported an abortion wave, nor was positive according to the 2008 or 2009 BTM programs, nor had positive vaginal swabs.

### Model description

**Figure 1:**
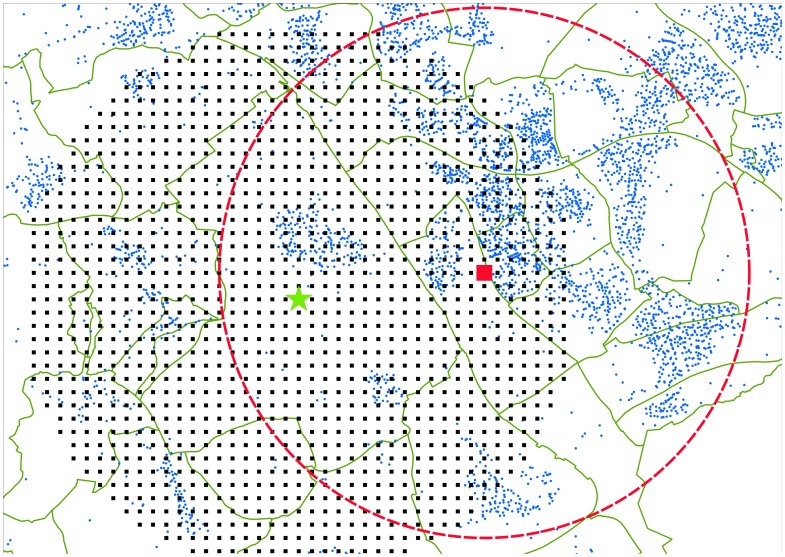
Overview of the data selection in outbreak area C. The PC4-polygons are indicated by the green lines. The center of the case cluster is indicated by the green star. This star is also the center of the spatial grid with a resolution of 250×250 m (black squares). Around each of the grid points (example indicated by the large red square) the distance *r* to all PC6's (small blue dots) within *Z* = 5000 m is determined, as well as the number of cases *k* and inhabitants *n* in these PC6's.

We defined the center of each outbreak area as the coordinates of the center of gravity of a four-digit zip code (PC4, i.e. at neighborhood-level) polygon with the highest incidence. The incidences in areas A, B and C were equal to 270, 540 and 1280 cases per 100 000 persons respectively (the national incidence in 2009 was 14 per 100 000 persons). The center coordinates were used as the center of a spatial grid (Figure 1). Each grid point represents an area of 250×250 m and is located in the center of this 250×250 m square.

Every individual grid point *j* represents the location of a putative source. We collected information from every PC6 *q* (with *q* = 1 … *Q*_j_) within *Z* = 5000 m [15], [19] around each point *j*: the number of inhabitants (*n_q,j_*) and cases (*k_q,j_*), and the distance (*r_q,j_*) from grid point *j* to PC6 *q*. The total modeling area thus has a radius of 10 km (i.e. the radius of the spatial grid plus *Z*), in which the total number of cases in 2009 was 106 (A), 278 (B) and 230 (C) respectively.

We performed a sensitivity analysis on the case selection radius *Z* (see supporting information in Text S1 and Figure S1), suggesting that *Z* = 5000 m is an appropriate choice. In case of a too low Z (≤3000 m) the number of cases and inhabitants is too low for reliable results; in case of a too high Z (≥7500 m) a too large area is depicted as a possible location of the putative sources.

We assumed that for each PC6 *q* within 5000 m of grid point *j* the number of cases *k_q,j_* is a realization from a binomially distributed random variable with parameters *p_q,j_* and *n_q,j_*, with *p_q,j_* being the probability of becoming ill due to *C. burnetii* in PC6 *q* due to a putative source in grid point *j*:
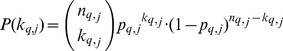
(1)According to spatial concentration theories (e.g., the Gaussian plume equation), concentrations around spatial point sources decrease exponentially as function of distance [20]. Also, since the incidences in the total population are relatively small, we assume the doses were relatively small and thus the relation between the incidence and the dose is approximately linear [21]. Hence, the risk of infection – being proportional to the concentration of a pathogen – decreases exponentially by distance from a source. Thus, we define the risk of becoming ill due to a putative source at grid point *j* as:
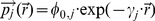
(2)for a baseline infectivity *φ_0,j_* [-] and decay parameter *γ_j_* [m^−1^]. Vector 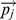 represents the probabilities of infection in all PC6's; vector 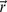 contains the distances from grid point *j* to all PC6's. For each grid point *j* we tested whether
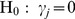
(3a)or
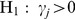
(3b)that is, whether the incidence-distance relationship is either constant (null hypothesis) or exponentially decreasing (alternative hypothesis). The parameters *φ_0,j_* and *γ_j_* are estimated by maximizing the log-likelihood *l_j_* for grid cell *j*:
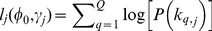
(4)which results in values for *φ_0,j_* and *γ_j_*. The parameters which maximize the log-likelihood in equation (4) were computed using R (version 2.15.1) [22] and the script is provided in the supporting information (Text S2).

Subsequently, the deviance is calculated as a measure for the difference between log-likelihood of the models of the null and alternative hypotheses:
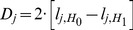
(5)Since the model of the alternative hypothesis is nested in the model of the null hypothesis, *D_j_* is *χ^2^*-distributed with one degree of freedom. Any grid point with *D_j_* significant at the 95% confidence level was classified as “exponential”, and thus representing a location for a putative source. All other grid points were classified as “constant”, and thus not representing a location for a putative source.

To compare the exponential grid points, we defined a *measure of risk* (MR), by integrating equation 2 from 0 to *L* = 2000 m [15]:
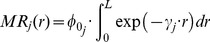
(6)A sensitivity analysis of the upper integration limit (Text S3 and Figure S2) suggested that *L* = 2000 m is an appropriate choice, since it not only gives a reasonable contrast of the hotspot with its surrounding, but also it is a regular distance from the suspected farms to nearby villages. Furthermore, Schimmer et al. (2010) [15] concluded that the risk for a human Q fever infection was highest within 2000 m from an infected farm.

Subsequently, we normalized the values of vector 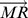 to values between 0 and 1, leading to a *normalized measure of risk* (nMR):
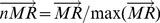
(7)and defined a *hotspot* as the collection of grid points in space with nMR>0.9. This value is chosen as it results in hotspots with a radius of ±1 km. If a lower limit is used, the radius of the hotspots increases to several kilometers.

The values of 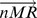 are based on the total list of notified cases in 2009 in each area. In addition, a time-dependent analysis was elaborated to determine the earliest week where the location of the final hotspots was identified. That is, we calculated the distance between the coordinates of the grid point with nMR = 1 of the *temporal* and the *final* hotspots and recorded the first week of stabilization of this difference by visual inspection. Finally, we defined temporal nMR-fractions as the sum of the elements of 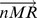 of a particular week (using the list of notified cases up to that week) divided by the sum of the elements of the final 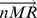 (using the total list of notified cases in 2009).

### Validation

For the validation analysis, all farms in the spatial grid were regarded as putative sources. We primarily focused on large farms (>50 animals), firstly because this was the standard for BTM-tests, and secondly because larger farms are supposedly associated with higher numbers of infections [8], [23]. This analysis was repeated including small (i.e. ≤50 animals) farms.

The likelihood of a putative source to be the actual source was determined by retrieving the nMR-value of the closest grid point. We then ranked the farms by their nMR-values and identified the ranking of the suspected farms.

### Multiple source simulation

A two-source simulation was performed to learn how the model behaved in case of more than one source in an outbreak area. Two artificial sources were added to a non-urban area in outbreak area C (although any other location in the Netherlands could be used as well). This allocation was semi-random, as we selected runs with a varying distance between the two sources.

For every source location, we generated cases as realization of a binomial distribution using the underlying population data at the PC6-level. The probability of infection in PC6 *q* from sources *s_1_* and *s_2_* is equal to:
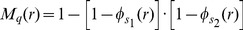
(8)for which we applied 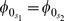
≈3.14×10^−2^ and 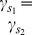
≈7.18×10^−4^ m^−1^, i.e. the mean baseline infectivity and the mean decay rate of the suspected farms in areas A, B, and C (values retrieved from the results section). Equation 8 gives us the probability of being infected at distance *r* by either source 1, or source 2, or both. With the spatial distribution of 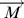 as input, the model needs to identify the location of the allocated sources. Approximately 250–300 binomial simulations were necessary for steady-state results of 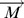, monitored by the running average of the elements of 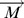, but to be on the safe side we performed 500 simulations per run. The size of the spatial grid was 10×10 km; its resolution was reduced to 500×500 m due to computational speed limitations. Cases were drawn from an area up to 5000 m outside the spatial grid.

## Results

### Area A

#### Non-temporal analysis

**Figure 2:**
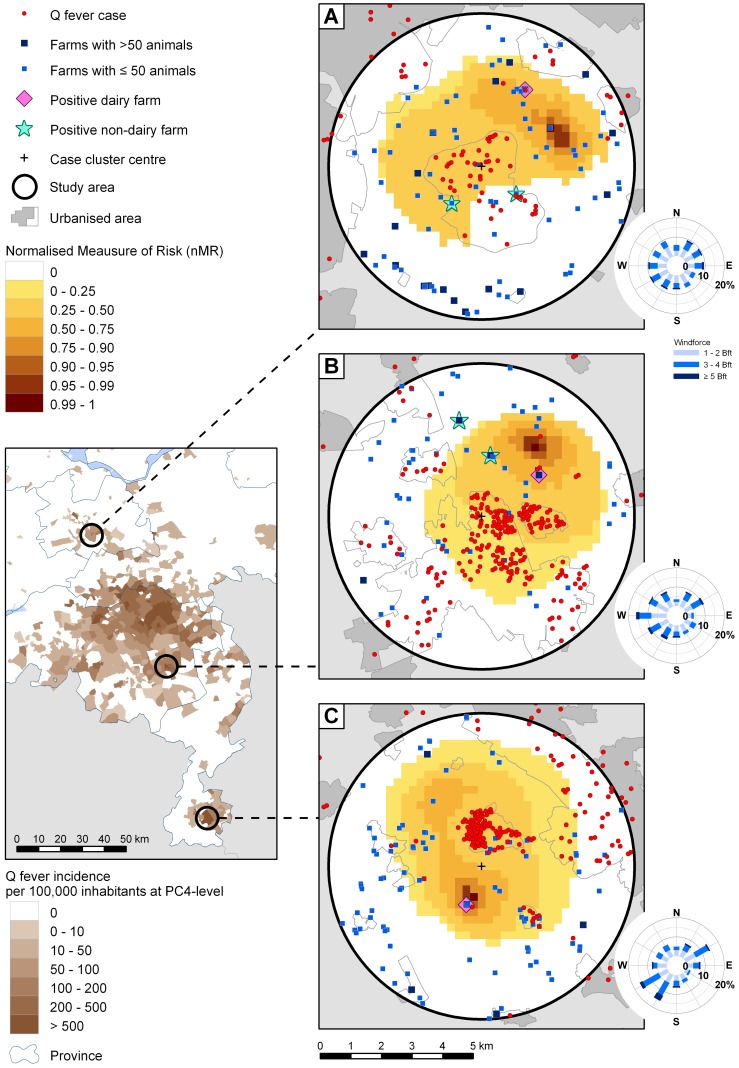
Maps of the normalized measures of risk (nMR) of the areas A, B, and C with a grid radius of 5000 m. Results are based on all cases in 2009. Diamonds indicate suspected farms; positive non-dairy farms are indicated by a star. Hotspots are the areas with a nMR≥0.9. For completeness, wind plots of the weeks 15–25 (area A), 11–17 (B), and 11–23 (C) are added. These are the weeks when approximately 90% of the cases was infected, corrected for an incubation period of 20.7 days for Q fever [31].

Figure 2 shows 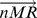 spatially represented by square grid cells. In area A, the hotspot is located 2200–3300 m northeast of the case cluster centre. Table 1 shows the nMR-value of the closest grid point from the suspected farm, being 0.56. Although the distance between the suspected farm and the hotspot is nearly 1500 m, the suspected farm is ranked as the most likely source out of 18. Taking into account small farms as well, the suspected farm is ranked as 12 out of 86. Furthermore, of the two non-dairy farms in the region, one is located near a grid point with nMR = 0.29; the other is located near low ranking exponential grid points and some constant grid points.

**Table 1:** Status information and number of goats/sheep of all dairy and non-dairy farms within the spatial grids of the areas A, B, and C that either reported abortions >5% [9] (“AB”), or that were tested either in the BTM programs in 2008 and 2009 [8], [10] (“BTM08”, and “BTM09”), or in the non-systematical vaginal swab program [11], [12] (“VS”).

Area	Type	# Goats	# Sheep	nMR-value	Test	Status
A	Dairy farm^a^	1295	-	0.56	BTM08, BTM09, VS	Positive
	Non-dairy farm	3	11	0.29	VS	Positive
	Non-dairy farm	5	14	-	VS	Positive
B	Dairy farm^a^	791	-	0.57	AB, VS, BTM08, BTM09	Positive
	Non-dairy farm	-	175	0.45	VS	Positive
	Non-dairy farm	-	133	-	VS	Positive
C	Dairy farm^a^	1135	-	0.89	AB, BTM08, BTM09	Positive
	Dairy farm	1325	-	-	BTM08, BTM09	All negative

Also, the nMR-value of the closest grid point is listed. Farms located near a constant grid point do not have a nMR-value.

a Suspected farms.

#### Temporal analysis

**Figure 3:**
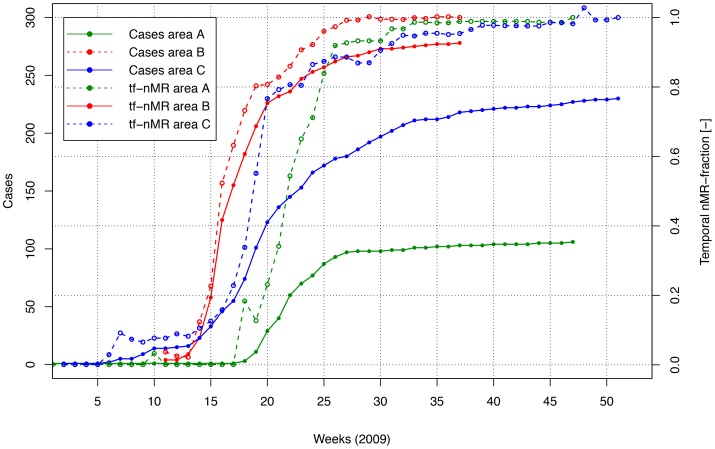
Cumulative number of cases per week (solid lines) and the temporal nMR-fraction (“tf-nMR”) (i.e. the spatial cumulative nMR-values per week as fraction of spatial cumulative nMR-values using all cases of 2009) (dashed lines) for the areas A (green), B (red), and C (blue).

Figure 3 shows the cumulative number of cases per week and the temporal nMR-fractions. The largest increase in the temporal nMR-fractions occurs in week 22, when the increase in cases is largest. The distance between the temporal and final hotspots declines very sharply in time and stabilizes below 1000 m in week 19 with 11 cases (10%) notified (not shown).

### Area B

#### Non-temporal analysis

The hotspot is located 2500–3500 m north of the case cluster centre at 900 m from the suspected farm. This farm is located near a grid point with nMR = 0.57 and is ranked as the most likely source out of four large farms. Taking into account the small farms as well, it is ranked as the third most likely source out of 56. One non-dairy farm in the region is located near a grid point with nMR = 0.45; the other is located near no high ranking exponential grid points and some constant grid points.

#### Temporal analysis

The largest increase in the temporal nMR-fraction occurs when the increase in cases is largest (i.e. week 16). The distance between the temporal and final hotspots stabilises from week 14 with 23 cases notified (8%).

### Area C

#### Non-temporal analysis

The hotspot is located 600–1600 m south of the case cluster centre at 300 m from the suspected farm, which is located near a grid point with nMR = 0.89. It is ranked as the most likely source out of five large farms. Including also the small farms, it remains the most likely source out of 97.

Furthermore, there is also a negative large dairy goat farm in the area, but that farm is located near low ranking exponential grid points and some constant grid points.

#### Temporal analysis

The increase of the temporal nMR-fraction is largest when the increase in cases is largest (week 19). The distance between the temporal and final hotspots stabilizes from week 8, following the first five notified cases (2%).

### Multiple source simulation

**Figure 4:**
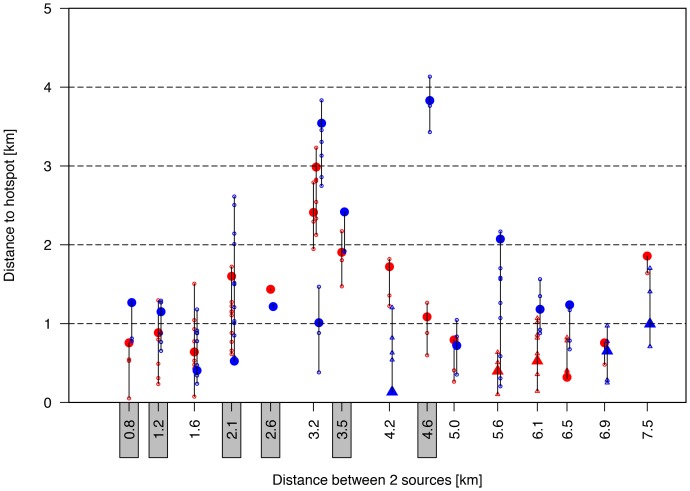
Two source simulations with the distance from the two sources to their closest hotspot as function of the distance between the two sources. The one source is indicated in red; the other in blue. The distance to all grid cells with nMR>0.9 is listed by small open circles; the grid cell with the maximum nMR-value in the hotspot is indicated by a large closed circle. If a local maximum with nMR-values<0.9 appeared, then triangle symbols are used, taking into account all grid cells with nMR-values of 0.10 lower than the local maximum. If a source could not be attributed to a single hotspot, then the distance to both hotspots was indicated (e.g., at x = 3.2 km). Results with just one hotspot are indicated by the grey rectangles at the x-axis.

Figure 4 shows the distance between the sources and the hotspots as function of the distance between the two sources. If from visual inspection it was unclear which hotspot belonged to which source, the distance to both hotspots was determined. If only one hotspot appeared (grey labels at x-axis), then the distance from both sources to that hotspot was determined.

In principle, a hotspot was defined as the area with nMR>0.9. However, since we normalized 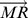, a second hotspot could be characterized by nMR<0.9. Therefore, we traced local maxima visually and depicted these with triangles in Figure 4.

The results in Figure 4 show that source detection is most clear if the distance between the sources is either relatively small or relatively large. That is, if the two sources are located close to each other, they are in general detected as one common source. If, on the other hand, they are located far from each other, they are in general detected as two individual sources. For intermediate distances, the confidence of detection is lower.

## Discussion

### General

We developed a model to identify the most likely source in an airborne infectious disease outbreak area based on human cases. From a public health perspective, it is desirable to identify the actual source rapidly, not only to reduce source-to-human transmission, but also to reduce the risk of source-to-source transmission. As a case study, we retrospectively analyzed three Q fever outbreaks in the Netherlands in 2009, with suspected exposure from a single large dairy goat farm in each area.

Distances from the hotspots to the suspected farms varied from 300–1500 m. The suspected farms were calculated as the most likely source out of 18 (area A), 4 (area B), and 5 (area C) large farms.

Regarding the time series results, the temporal nMR-fractions show the largest increase during the week with the highest increase of new notifications. The distance between the temporal and final hotspots declines rapidly in time and stabilizes below 1000 m when 10% (area A), 8% (area B), and 2% (area C) of the cases had been notified.

The two-source simulations show that source detection is most clear if the distance between the sources is either relatively small or relatively large. That is, if the two sources are located close to each other, they are in general detected as one common source. If they are located far from each other, they are in general detected as two individual sources. For intermediate distances, in general one of the two sources is identified and the other is not, possibly caused by ‘overlapping’ incidence curves resulting in a less clear source detection.

Our model does not require input of wind speed and wind direction data since it is a radially-symmetric model: it selects the data uniformly in all directions within a chosen radius. In our study, we used a radius of *Z* = 5000 m (see supporting information in Text S1 and Figure S1), but it might differ according to specific airborne pathogen (e.g., the Highly Pathogenic Avian Influenza Virus [24]). Furthermore, it might be necessary to increase the radius under certain environmental conditions, such as high wind speeds from one prevailing direction. In our study however, the wind direction was very variable during the weeks with most notified cases in all three areas (see wind plots in Figure 2).

In addition, further information may improve the model, such as land-use characteristics. In area B, for instance, a forest is situated between the suspected farm and the calculated hotspot. High vegetation densities promote the deposition of particles, thereby reducing the probability that the actual source was situated north of the forest.

Possible applications for the model do not only include outbreaks of airborne pathogens from livestock and industries (e.g., the foot-and-mouth disease virus and Legionalla bacteria), but also deliberate pathogen releases, e.g., a release of *Bacillus anthracis* (anthrax) as a biological weapon.

### Animal factors (exposure)

Next to source-to-human transmission, there is also the risk of source-to-source transmission [25], which in the Dutch outbreaks may have contributed to the final large number of positive dairy goat and sheep farms. However, from the reported abortions and BTM-results in 2008 and 2009, we assumed there were no additional large goat farms infected in the areas under consideration.

The lists of farms with positive vaginal swabs [11], [12] also contained non-dairy farms. However, a difficulty is that these tests were performed non-systematically, i.e. only a fraction of the non-dairy farms were tested. Also, individual goat vaginal swab sampling is not directly comparable to BTM sampling, due to differences in sensitivity, subject representation (BTM samples represent whole populations), and sampling period.

From our results, infection from non-dairy farms was in general less likely to have played a role. In area A, one non-dairy farm is located near a grid point with a low nMR-value, and the other one is located near no high ranking exponential grid points and constant grid points. The latter is also true for one of the non-dairy farms in area B. The other non-dairy farm in area B might have played a role, since it is located close to the high-risk zone.

Formal validation of the model in terms of sensitivity and specificity is not possible, since in general only dairy farms with >50 goats/sheep were tested and the majority of the farms in the outbreak areas are small non-dairy farms. Nevertheless, by visual inspection, and considering farm characteristics (number of animals, positivity), we believe that the model is capable of allocating regions where the actual source is most likely located.

Sampling all sources in an outbreak area is expensive and time-consuming. However, initially testing the sources within or near a hotspot as indicated by the model is much more feasible, and may thus contribute to a possible early detection of the actual source.

### Human factors (risk)

In the current study we used the residential addresses of notified cases as a proxy for the location of exposure. The airborne transmission route might have been either direct (i.e. bacteria emitted from a source are directly transmitted to a person) or indirect (i.e. emitted bacteria are first deposited in the environment and transmitted at a later moment due to re-emission). In our approach, basically both routes are incorporated since we only take into account farm and case notifications. This has great advantages, since the exact time and place of infection is unknown. Although Dutch people spend about 70% of the day at home [26], they could have been infected outdoor, for instance when cycling to work or school, during recreation, or at work. Environmental contamination of *C. burnetii* is possible [27], [28], although it is also said to be a minor contributor with respect to direct airborne transmission [29].

Also, our model does not include information about risk factors like socioeconomic status or smoking behavior. Dijkstra et al. (2012) [5] concluded that men, smokers and people aged 40–60 years were at increased risk. We did not use data on the spatial distribution of smokers, gender, and age.

The decrease in notified cases in 2010 compared to 2009 may be an effect of reduced exposure (vaccination and culling) [13], but also of an increased immunity in the general population [5]. This might influence the effectiveness of the model in future Q fever outbreaks in the Netherlands, as the number of susceptible people might have decreased.

Our model is more robust than other epidemiological tools such as circular ring attack rate analyses [30], as it does not make use of coarse discretization methods. Therefore, it is less sensitive to relatively low incidences. Also, we have applied formal statistical criteria to determine the likelihood of a farm to be the actual source, whereas these formal criteria are absent in concentric ring analyses.

Finally, the method is usable for real-time monitoring systems both to automatically detect outbreak areas and to use it as a first detection tool before taking samples from putative sources and their surrounding environments. This way not only time can be gained, but also costs and the number of human and source-to-source infections can be reduced.

## Conclusions

Using the data from three Q fever outbreak areas in the Netherlands in 2009, the suspected sources are identified as the most likely source of infection with hotspots at 300–1500 m. The areas of high risk (hotspots) are detected in the early stages of the outbreaks. The method is applicable to other comparable airborne pathogens, although for each pathogen a sensitivity analysis should be performed on the spread distance (*Z*) and the integration distance (*L*). Also, a possible pathogen inactivation rate should be incorporated.

Our model is more robust than other epidemiological tools such as circular ring attack rate analyses, as it does not make use of coarse discretization methods. Therefore, it is less sensitive to low case numbers. Furthermore, our method uses a case-to-source approach and no wind data are used, whereas Gaussian dispersion models are generally based on a source to case approach using wind measurements. Also our method is usable for real-time monitoring systems to both detect outbreak areas automatically and to use as a first detection tool before taking samples from putative sources and their surrounding environments. Thus time can be gained, and costs and the number of human and source-to-source infections can be reduced.

## Supporting Information

Text S1 **Sensitivity analysis on the case selection radius** ***Z*** **.**
(DOCX)

Text S2 **R-script for maximizing the log-likelihood (** **equation 4** **).**
(PDF)

Text S3 **Sensitivity analysis on the upper integration limit** ***L*** **in** **equation 6** **.**
(DOCX)

Figure S1
**Average nMR as function of the distance to the hotspots in area A, B and C for different values of the selection radius** ***Z*** **: 1, 2, 3, 4, 5, 7.5, and 10 km.** The average nMR is retrieved by applying a loess-function (in R, version 2.15.1) to all 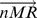 values per area as function of the distance of each grid point to the hotspot in that area.(TIF)

Figure S2
**Average nMR as function of the distance to the hotspots in area A, B and C for different values of the integration distance** ***L*** **: 0.1, 0.5, 1, 2, 3, 4, 5, 10, and 20 km.** The average nMR is retrieved by applying a loess-function (in R, version 2.15.1) to all 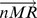 values per area as function of the distance of each grid point to the hotspot in that area.(EPS)
