# Supplementary material for: A Model for the Early Identification of Sources of Airborne Pathogens in an Outdoor Environment
Source: PLoS One. 2013 Dec 4;8(12):e80412. doi: 10.1371/journal.pone.0080412 (PMC3850919; doi:10.1371/journal.pone.0080412)
Supplement: Text S1 — Sensitivity analysis on the case selection radius Z . (DOCX) [file pone.0080412.s001.docx]

**Text S1: Sensitivity analysis on the case selection radius *Z***

**Problem definition**

A value of *Z* = 5000 m was chosen as the distance to which data on cases and population per PC6 was collected around each grid point. This value is based on results of [15], who concluded that the majority of the Q fever cases lived within 5000 m of an infectious goat farm. This supporting material shows a sensitivity analysis on this value in the perspective of the source detection method.

**Method**

The sensitivity analysis was performed for the non-temporal model situation in the three outbreak areas by using *Z* = 1, 2, 3, 4, 5, 7.5 and 10 km. The resulting $\overrightarrow{nMR}$ was put in a loess-smoothing model (function loess() with span = 0.2, in package ‘stats’ in R, version 2.15.1), as function of the distance to the reference grid point with nMR = 1 in the situation with *Z* = 5000 m. The x and y-coordinates of this reference grid point in areas A, B and C are [142285, 450767], [176026,391742] and [192670, 319045] respectively in EPSG Projection system 28992.

**Results**

Supplementary Figure S1 shows the smoothed nMR-values as function of the distance from the reference grid points for the different values for *Z*. In general, if *Z* is low (i.e. 1000 and 2000 m in all areas, and also 3000 m in area A) the hotspots are not detected and the spatial distribution of $\overrightarrow{nMR}$ does not show a coherent pattern (not shown). This is shown as the incomplete lines in Figure S1. The higher *Z*, the higher the length of $\overrightarrow{nMR}$, i.e. the more grid points are classified as exponential. The hotspots are still detected correctly, but the contrast with their surrounding decreases (higher values in Figure S1). This occurs mainly for *Z* ≥ 7500 m.

Note the bump in area C at approximately x = 3500 m, which is basically the second minor hotspot in the northwestern part of the outbreak area (see Figure 2). This is only detected for values of *Z* = 3, 4 and 5 km.

**Conclusions**

The results show that if *Z* is relatively small (≤ 3000 m), the hotspots are not detected and the spatial distribution of $\overrightarrow{nMR}$ does not show a coherent pattern. If *Z* is large (≥ 7500 m) the contrast of the hotspots decreases and the number of grid points classified as ‘constant’ decreases. In combination with the findings of [15], we think it is plausible to use *Z* = 5000 m in the model.
