# Supplementary material for: A Model for the Early Identification of Sources of Airborne Pathogens in an Outdoor Environment
Source: PLoS One. 2013 Dec 4;8(12):e80412. doi: 10.1371/journal.pone.0080412 (PMC3850919; doi:10.1371/journal.pone.0080412)
Supplement: Text S2 — R-script for maximizing the log-likelihood ( equation 4 ). (PDF) [file pone.0080412.s002.pdf]

Text S2: R-script for maximizing the log-likelihood (equation 4)

```
############################ GENERAL FUNCTIONS
ilogit <- function( x ) {
  return( 1 / ( 1 + exp( -x ) ) )
}
transform <-function( theta ) {
  return( c( ilogit( theta[ 1 ] ), exp( theta[ 2 ] ) ) )
}



############################ OPTIMALISATION OF ALPHA AND GAMMA
optimalisation <- function( n, k, r ) {
  # INPUT:
  # -n: Array of number of inhabitants per PC6 q within 5000 m of source j
  # -k: Array of number of cases per PC6 q within 5000 m of source j
  # -r: Array of distances to all PC6's within 5000 m of source j



  # Likelihood function
  # 'theta' is a parameter vector, theta[i] is always between -Inf and +Inf
  # In null hypothesis: theta <- c( alpha_init, gamma_init)
  # In alternative model: theta <- alpha_init;
  lik.fun <- function( theta ) {
    if ( length( theta ) == 1 ) {
      gamma                     <- 0
    } else gamma                <- transform( theta )[ 2 ]
```

```
32      alpha_beta                  <- transform( theta )[ 1 ]
33      p                           <- alpha_beta * exp( -gamma * r )
34      result                      <- -sum( dbinom(x = k, size = n,
35                                                  prob = p, log= T ) )
36      return(neg.log.lik)
37    }
38
39    # OPTIMALISATION OF THETA
40    # Runs as long as the determinant > 0.
41    criterium                     <- -10
42    while( criterium <= 0) {
43      beta.init                   <- rnorm( n= 1, mean= 0, sd= 1 )
44      alpha.init                  <- beta.init
45      gamma.init                  <- rnorm( n= 1, mean= -8, sd= 2 )
46
47      lik.opt.const               <- try( optim(par = beta.init,
48                                                fn = lik.fun,
49                                                method = "BFGS",
50                                                hessian = TRUE),
51                                          silent = T )
52      lik.opt.exp                 <- try( optim(par = c( alpha.init,
53                                                         gamma.init ),
54                                                fn = lik.fun,
55                                                method = "BFGS",
56                                                hessian = TRUE),
57                                          silent= T )
58      if ( is( lik.opt.const, "try-error") ||
59           is( lik.opt.exp, "try-error" ) ) next
60      criterium                   <- det( lik.opt.exp$hessian)
61    }
62
63
```

```
64    # Get data
65    with(lik.opt.exp, {
66      alpha_exp                    <<- transform( par )[1]
67      gamma_exp                    <<- transform( par )[2]
68      lik_exp                      <<- value
69    })
70
71    with(lik.opt.const, {
72      beta_lin                     <<- transform( par )[1]
73      lik_lin                      <<- value
74    })
75
76
77    # Save data in matrix
78    results                      <- matrix( data= c( alpha_exp,
79                                                     gamma_exp,
80                                                     lik_exp,
81                                                     beta_lin,
82                                                     lik_lin ),
83                                            nrow= 1, ncol= 5)
84    colnames( results )          <- c( "alpha_exp",
85                                       "gamma_exp",
86                                       "lik_exp",
87                                       "beta_lin",
88                                       "lik_lin")
89
90    return(results)
91  } # END OF FUNCTION 'optimalisation'
```
