# Supplementary material for: A Model for the Early Identification of Sources of Airborne Pathogens in an Outdoor Environment
Source: PLoS One. 2013 Dec 4;8(12):e80412. doi: 10.1371/journal.pone.0080412 (PMC3850919; doi:10.1371/journal.pone.0080412)
Supplement: Text S3 — Sensitivity analysis on the upper integration limit L in equation 6 . (DOCX) [file pone.0080412.s003.docx]

**Text S3: Sensitivity analysis on the upper integration limit *L* in equation 6**

**Problem definition**

In equation 6 an upper integration limit *L* = 2000 m was used to determine the Measure of Risk per grid point. This supplementary material shows a sensitivity analysis on this upper integration limit. We initially hypothesised that *L* would ideally be approximately 2000 m for three reasons. Firstly, it might occur that the MR-value of a grid point that is *just* classified as exponential instead of constant is much higher than the MR-value of a “very” exponential grid point, if the integration distance is too large. In other words: the former has small values for *ϕ_0_* and *γ*; the latter has high values for *ϕ_0_* and *γ*. Secondly, 2000 m is a regular distance from the actual putative sources to the nearby municipality and the majority of the cases. Thirdly, Schimmer et al. (2010) [15] concluded that cases living within 2000 m from an infected farm are most at risk compared to those living at 5-10 km from an infected farm.

**Method**

This sensitivity analysis was performed for the non-temporal model situation by using *L* = 0.1, 0.5, 1.0, 2.0, 3.0, 4.0, 5.0, 7.5, 10, and 20 km. The resulting $\overrightarrow{nMR}$ was put in a loess-smoothing model (function loess() with span = 0.2, in package ‘stats’ in R, version 2.15.1), as function of the distance to the reference grid point with nMR = 1 in the situation with *L* = 2000 m. The x and y-coordinates of this reference grid point in areas A, B and C were [142285, 450767], [176026,391742] and [192670, 319045] respectively in EPSG Projection system 28992.

**Results**

Supplementary Figure S3 shows the smoothed (averaged) nMR-values as function of the distance from the reference grid points for the different values for *L*. The hotspots are detectable in all areas for all values of *L*, since all curves originate from [0,1]. Nevertheless, the contrast of the hotspots with the surrounding grid points decreases as *L* increases. Note that the nMR-values in area B tend to increase for *L* = 10 km or *L* = 20 km. This is due to first consideration mentioned in the problem definition.

**Conclusions**

Concluding, the lowest value for *L* (100 m) gives the largest contrast of the computed hotspot with its surrounding. However, since there are always uncertainties in a model, it is desirable to have a not too high contrast, i.e. the hotspot area (nMR > 0.9) is smaller for *L* = 100 m than for *L* = 20 km. In combination with the second and third considerations as mentioned in the background text, we think it is very plausible to use the 2000 m as the preferred upper integration limit.
