# Supplementary figures and images for: A Model for the Early Identification of Sources of Airborne Pathogens in an Outdoor Environment
Source: PLoS One. 2013 Dec 4;8(12):e80412. doi: 10.1371/journal.pone.0080412 (PMC3850919; doi:10.1371/journal.pone.0080412)

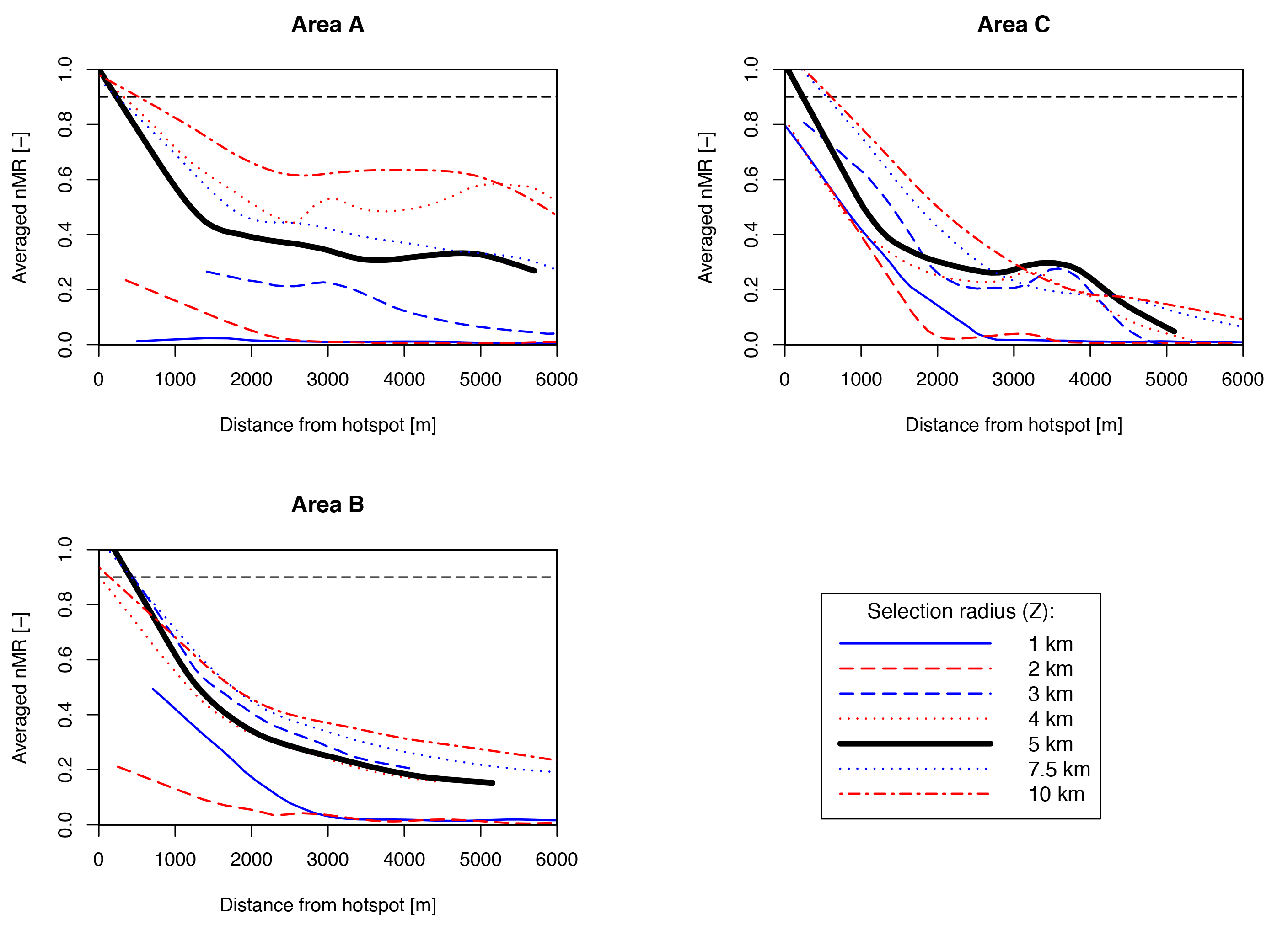

Supplement: Figure S1 — Average nMR as function of the distance to the hotspots in area A, B and C for different values of the selection radius Z : 1, 2, 3, 4, 5, 7.5, and 10 km. The average nMR is retrieved by applying a loess-function (in R, version 2.15.1) to all values per area as function of the distance of each grid point to the hotspot in that area. (TIF) [file pone.0080412.s004.tif]
